# A novel cancer/testis antigen KP-OVA-52 identified by SEREX in human ovarian cancer is regulated by DNA methylation

**DOI:** 10.3892/ijo.2012.1508

**Published:** 2012-06-06

**Authors:** KANG-MI KIM, MYUNG-HA SONG, MIN-JU KIM, SAYEEMA DAUDI, ANTHONY MILIOTTO, LLOYD OLD, KUNLE ODUNSI, SANG-YULL LEE

**Affiliations:** 1 Departments of Microbiology and Immunology and; 2 Biochemistry, School of Medicine, Pusan National University, Yangsan-si, Gyeongsangnam-do 626-770, Republic of Korea;; 3 Department of Gynecologic Oncology and Center for Immunotherapy Roswell Park Cancer Institute, New York, NY 142634;; 4 Ludwig Institute for Cancer Research, New York Branch at Memorial Sloan-Kettering Cancer Center, New York, NY 10021, USA

**Keywords:** serologic analysis of recombinant cDNA expression, cancer/testis antigen, ovary cancer, DNA methylation

## Abstract

SEREX has proven to be a powerful method that takes advantage of the presence of spontaneous humoral immune response in some cancer patients. In this study, immunoscreening of normal testis and two ovarian cancer cell line cDNA expression libraries with sera from ovarian cancer patients led to the isolation of 75 independent antigens, designated KP-OVA-1 through KP-OVA-75. Of these, RT-PCR showed KP-OVA-52 to be expressed strongly in normal testis, in ovarian cancer cell lines (3/9) and in ovarian cancer tissues (1/17). The expression of KP-OVA-52 in cancer cells is also induced by the demethylating agent 5-aza-2′-deoxycytidine (ADC). To test immunogenicity, we used the Serum Antibody Detection Assay (SADA) to analyze anti-IgG antibodies against the 75 antigens that were initially isolated by SEREX. Four of the 75 antigens (KP-OVA-25, KP-OVA-35, KP-OVA-68 and KP-OVA-73) reacted exclusively with sera from cancer patients. However, KP-OVA-52 reacted with 1 of 20 ovarian cancer sera. These data suggest that the KP-OVA-52 can be considered a novel CT antigen that is regulated by DNA methylation.

## Introduction

Ovarian cancer is the leading cause of death from gynaecological malignancies (1,2). It accounts for 5% of all cancer deaths among women with an estimated 21,880 new cases and 13,850 deaths from ovarian cancer in the United States in 2010 (2). The poor prognosis and high mortality rate associated with the disease have not significantly improved over the last 30 years despite advances in treatment (3). Current therapies are effective for patients with early stage disease (FIGO stage I/II) where 5-year survival rates range from 73% to 93%. Their usefulness, however, is limited for patients with advanced stage disease where the 5-year survival is only about 30% (2). Because so many ovarian cancer patients are diagnosed at a later stage, it is important to find methods by which to improve treatments for more advanced disease.

The selective recognition of tumor antigens by the immune system provides a powerful means to screen for tumor-associated antigens (TAAs). The identification of tumor antigens has yielded an array of target molecules for diagnosis, monitoring, and immunotherapy of human cancer (4). The serologic analysis of recombinant cDNA expression libraries (SEREX) was designed to combine serologic analysis with antigenic cloning techniques to identify human tumor antigens eliciting high-titered IgG antibodies (5) and has provided a powerful approach to identify immunogenic tumor antigens. To date, over 2,500 tumor antigens have been identified from a variety of malignancies using SEREX, which include gastric cancer (6), colon cancer (7), melanoma (8), breast cancer (9), renal cell carcinoma (10), lung cancer (11) and leukemia (12). These antigens can be classified into several categories, including mutational antigens (5,10), differentiation antigens (10,14), over-expressed antigens (15) and cancer/testis (CT) antigens (16,17). CT antigens are the products of transcripts present only in developing germ cells and human cancers of diverse origins that elicit spontaneous cellular and humoral immune responses in some cancer patients (18,19). Because of their tissue-restricted expression and immunogenicity, CT antigens are potential targets for vaccine-based immunotherapy (20).

Previous SEREX analysis of ovarian cancer by Stone *et al* (21) resulted in the detection of 25 distinct antigens. The majority of these antigens were recognized only by autologous serum, however 6 antigens were found to be immunogenic in at least 2 of the 25 patient sera. Additional studies on ovarian cancer have been performed by Luo *et al* (22) and Lokshin *et al* (23), who identified 12 and 20 ovarian cancer associated antigens, respectively. OVA-66 antigen identified Jin *et al* (24) was assessed for immunogenicity by ELISA using 48 control sera and 113 cancer sera from patients with various malignancies including ovarian cancer. OVA-66 reacted with 6 out of 27 sera from ovarian cancer patient (22.2%). The homeobox genes HOX-A7 and HOX-B7 (25,26) reacted with serum samples from 16/24 (66%) and 13/39 (33%) ovarian cancer patients, respectively, while normal individuals showed little or no reactivity toward these antigens. Expression of these gene products is not tissue-restricted at the mRNA level, and it is therefore unlikely that these antigens represent viable vaccine targets. These SEREX-defined ovarian cancer related antigens were known as TAAs but were not found to be significant CT antigens.

In the present study, the SEREX methodology was applied to further define the spectrum of immunogenic proteins in serous ovarian cancer patients. A specific focus was given to the KP-OVA-52 gene to determine its potential as a possible CT antigen.

## Materials and methods

### Human tissues, sera and cell lines

Human tumor tissues and sera were obtained from the Department of Gynecologic Oncology, Roswell Park Cancer Institute and Department of Pathology, Pusan National University Hospital after diagnosis and staging. The tissues were frozen in liquid nitrogen and stored at −80°C until use. Human ovarian cancer cell line SK-OV-3; human colon cancer cell lines SNU-C1 and SNU-C2A; human lung cancer cell lines SK-LC-5 and SK-LC-14; human breast cancer cell line MCF7; and human small cell lung cancer cell lines NCI-H82, NCI-H146, and NCI-H189 were obtained from the Korean Type Culture Collection and the American Type Culture Collection. All these cell lines were maintained in RPMI-1640 (Gibco-BRL Life Technologies Inc., Grand Island, NY, USA) medium supplemented with 10% fetal bovine serum (FBS), 2 mM L-glutamine, 100 U/ml penicillin and 100 *μ*g/ml streptomycin.

### Total-RNA extraction from tissues and cell lines

Total-RNA was isolated from human tissue samples and human tumor cell lines using the standard TRIzol reagent (Life Technologies, Gaithersburg, MD, USA) and RNA isolation kit (RNeasy maxi kit, Qiagen) following manufacturer’s instructions. The amount of isolated RNAs was measured by spectrophotometer (Ultrospec 2000, Pharmacia Biotech) at 260 nm. Normal tissue total-RNAs were purchased from Clontech Laboratories Inc., (Palo Alto, CA, USA) and Ambion Inc. (Austin, TX, USA).

Total-RNA from various cell lines were obtained from the cell repository of the Ludwig Institute for Cancer Research, New York Branch at the Memorial Sloan-Kettering Cancer Center and analyzed with reverse transcriptase-polymerase chain reaction (RT-PCR).

### Preparation of cDNA library and sera

Poly(A)^+^ RNA from normal testis was purchased from Clontech Laboratories Inc. and Poly(A)^+^ RNAs from SK-OV-3 and SNU 840 ovarian cancer cell lines were prepared by using the Fast Track mRNA purification kit (Invitrogen, Life Technologies, Carlsbad, CA, USA). mRNA 5 *μ*g was used to construct a cDNA library in the ZAP Express vector (Stratagene, La Jolla, CA, USA), following the manufacturer’s instructions. The library contained approximately one million recombinants and was used for immunoscreening without prior amplification. Five preparations of sera from ovarian cancer patients were used independently. The patients ages ranged from 45–64 years and all patients had advanced (stages III/IV) disease, with serous histology. Each serum sample was diluted 1:200 for SEREX analysis. To remove serum antibodies reactive with *E. coli*/bacteriophage-related antigens, sera were absorbed against *E. coli*/bacteriophage lysates as described by Lee *et al* (11).

### Immunoscreening

Immunoscreening of the cDNA library was performed as described (11,17). Briefly, *E. coli* XL1 blue MRF cells were transfected with the recombinant phages, plated at a density of approximately 5,000 pfu/150-mm plate (NZCYM-IPTG agar), incubated for 8 h at 37°C, and transferred to nitrocellulose filters (PROTRAN BA 85, 0.45 *μ*m, Schleicher & Schuell). Then the filters were incubated with a 1:200 dilution of the patient sera, which had been preabsorbed with *E. coli-*phage lysate. The serum-reactive clones were detected with AP-conjugated secondary antibody and visualized by incubation with 5-bromo-4-chloro-3-indolyl-phosphate/nitroblue tetrazolium (BCIP/NBT). After screening, the isolated positive clones were removed from the plate and conserved in suspension medium (SM) buffer with 25 *μ*l of chloroform. Positive phages were mixed with a helper phage to co-infect XL-1 Blue MRF, and they were rescued into pBluescript phagemid forms by *in vivo* excision. The excised phagemids were transformed into the host bacteria (XLOLR) to multiply for plasmid extraction and stock. The size of the inserted cDNA was determined primarily by double restriction enzyme digestion with EcoRI and XhoI. The cDNA was sequenced commercially (Macrogen, Seoul, Korea).

### RT-PCR

The cDNA preparations used as templates for RT-PCR reactions were prepared using 1 *μ*g of total-RNA in conjunction with the Superscript first strand synthesis kit (Invitrogen, Life Technologies). PCR primers were as follows: KP-OVA-52 5′/3′:AGGAGAAGCGGCAGAGTGGC/AGGTCCTTCCGTC TGGAGCG. KP-OVA-35 5′/3′:TGAGGAGGAGCAAGAGCC GA/TGGTGCTGTGTGAAGCACAC. KP-OVA-38 5′/3′:TGAT GACGCCACGTGGGCCG/GCTCTGGTGGCTTGGGTGGC. The cDNA templates were normalized on the base amplification of GAPDH. For PCR, 20 *μ*l reaction mixture consisting of 2 *μ*l cDNA, 0.2 mM dNTP, 1.5 mM MgCl_2_, 0.25 *μ*M gene specific forward and reverse primers, and 3 units Taq DNA polymerase (Solgent, Daejun, Korea) was preheated to 94°C for 5 min, followed by 35 cycles of 94°C for 30 sec, 60°C for 30 sec and 72°C for 1 min followed by a final elongation step of 72°C for 5 min. Amplified PCR products were analyzed on 1.5% agarose gels stained with ethidium bromide.

### Real-time quantitative RT-PCR

Oligonucleotide primers for the real-time quantitative PCR were synthesized commercially. PCR reaction mixtures (20 *μ*l), consisting of 2 *μ*l cDNA (or 2.0 *μ*l of genomic DNA amplification controls), 0.2 mM dNTP, 1.5 mM MgCl_2_, 0.25 *μ*M gene specific forward and reverse primers, and 2.5 units Platinum Taq DNA polymerase (Invitrogen, Life Technologies), were heated to 55°C for 5 min, 94°C for 10 min, followed by 40 thermal cycles of 94°C for 30 sec, 65°C for 30 sec and 54°C for 30 sec and a final cycle of 72°C for 30 sec. Thermal cycling was performed using a Perkin Elmer GeneAmp PCR System 9700. Resultant PCR products were analyzed in 2% agarose/Tris-acetate-EDTA gels. Primer sequences were as follows: KP-OVA-52 TaqMan primer: 5′-ACCAGCACTCAGAA CAACAGC-3′. KP-OVA-52 TaqMan primer: 3′-TCCTCCGAT GCCAGAAGAGTC-5′. KP-OVA-52 Taq Man probe: 5′-CTGC GTCAGTGAGGTCCTTCCGTCT-3′. The parameter Ct was defined as the threshold cycle number at which the fluorescence generated by cleavage of the probe exceeded the baseline. The target message was quantified by measuring the Ct value. GAPDH transcripts were quantified as endogenous RNA control using TaqMan human GAPDH control regents (Applied Biosystems).

### Serum antibody detection assay (SADA)

Phages corresponding to 48 antigens that were identified in SEREX screen were screened with individual sera by the SADA method as described by Lee *et al* (17). Briefly, 50 *μ*l of phages (500 pfu) were placed in duplicate wells of a 96-well microtitre plate. The phages were then transferred to a rectangle shaped agar plate covered previously with *E. coli* XL1-Blue MRF, top agarose and 10 mM IPTG by replica pin. The plates were incubated overnight at 37°C and were incubated with nitrocellulose transfer membranes for an additional 4 h. Membranes were used immediately for immunoscreening with each human serum.

### Generation of recombinant KP-OVA-52 fusion proteins

The open reading frame (ORF) cDNA inserts of the hyphotetical protein, KP-OVA-52, from gene bank (MN001042367), were subcloned into the pET23a expression plasmids containing a poly-histidine tag (Novagene). The expected protein size was about 29.5 kDa. The induction of recombinant protein synthesis by isopropyl β-D-thiogalactoside was performed at a low culture temperature (20°C). Protein synthesis was monitored by SDS/Poly-acrylamide gel electrophoresis and Coomassie Blue staining.

### Western blot analysis

About 100 ng of purified X6 His-proteins were separated on 10% SDS-PAGE and transferred onto nitrocellulose membranes (Hybond-ECL, GE Healthcare). After blocking with TBST (TBS, 0.1% Tween-20) containing 5% skim milk for 1 h at room temperature, the membrane was incubated in sera diluted to 1:200 overnight at room temperature. The membranes were washed and incubated with horseradish peroxidase-conjugated sheep anti-human IgG antibody (GE healthcare) diluted to 1:3,000 for 1 h at room temperature. After washing with TBST and mounting with the chemiluminescence reagent plus (Perkin Elmer), the membrane was exposed to Kodak medical X-ray film.

### Treatment of cells with ADC

The various cell lines were grown in RPMI-1640 (Life Technologies) and DMEM supplemented with 10% FBS in a 5% CO_2_ humidified atmosphere. In experiments designed to determine the effect of demethylation on the expression of KP-OVA-52 gene, actively replicating cells were treated with 1 *μ*M of ADC (Sigma, St. Louis, MO, USA) for 5 days. At the end of treatment, total-RNAs was purified and KP-OVA-52 gene expression was analyzed by RT-PCR.

## Results

### Identification of ovarian cancer antigens by SEREX

Immunoscreening of two ovarian cancer cell lines and testis cDNA expression libraries with the selected serum led to the isolation of 151 seroreactive cDNA clones. These clones include 75 independent antigens from testis and ovarian cancer cell lines, and were designated KP-OVA-1 through KP-OVA-75 (Tables I and II). When the cDNA sequences encoding the 75 ovarian cancer antigens were compared to those deposited in cancer immunome database (27). Forty-nine of the 75 antigens (65%) had been previously identified by SEREX analysis with any cDNA/serum combination whereas 26 (35%) have not been previously reported (Tables I and II). These 75 antigens comprise of 66 known proteins together with 9 uncharacterized gene products, which does not present in the sequences designated in the databases as expressed sequence tags (ESTs), KIAA series clones, FLJ series clones, MGC series clones, DKFZ series clones, and anonymous open reading frames (ORFs). The immunomic pattern of the tumor antigens that were previously identified in SEREX by other groups in the cancer immunome database was analyzed. One antigen (KP-OVA-40/MAGE10A) out of 49 previously identified antigens reacted with sera from ovarian cancer patients. The remaining 48 antigens are known to be associated with other tumor types including breast cancer, glioma, leukemia, melanoma, sarcoma, Hodgkin’s disease, breast, lung, hepatocellular, renal cell and prostate cancer. However, these antigens have not been known to be associated with ovarian cancers.

### Putative CT-like antigens and RT-PCR in normal tissues and cancer cell lines

**Figure 1:**
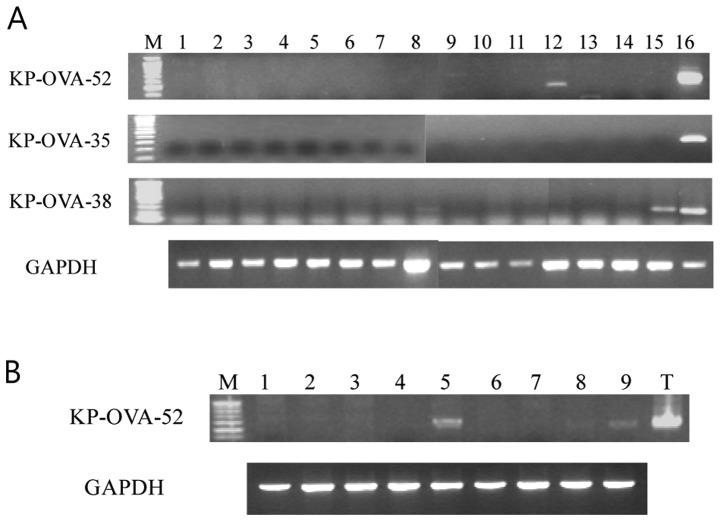
Conventional RT-PCR analysis of antigens in normal tissues and ovarian cancer lines. (A) From the left: 1, spleen; 2, thymus; 3, prostate; 4, ovary; 5, small intestine; 6, colon; 7, leukocyte; 8, heart; 9, brain; 10, placenta; 11, lung; 12, pancreas; 13, liver; 14, skeletal muscle; 15, kidney and 16, testis. The cDNA templates were normalized using GAPDH as shown in the bottom panel. (B) From the left: 1, A-10; 2, OV-2774; 3, OV-CAR-3; 4, SK-OV-1; 5, SK-OV-3; 6, SK-OV-4; 7, SK-OV-6; 8, SNU-8 and 9, SNU-840.

**Figure 2:**
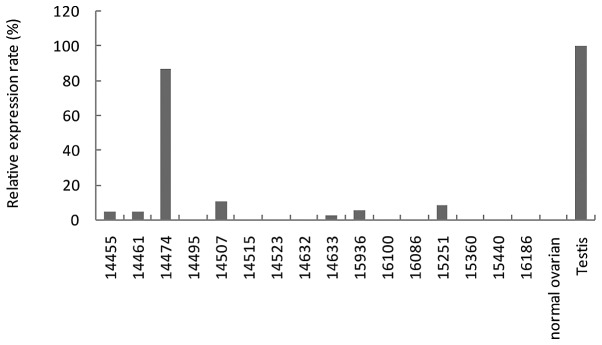
Quantitative real-time RT-PCR analysis of mRNA expression in ovarian tumors. Overexpression in cancer tissues was defined as 10% higher expression than the expression in normal tissue, excluding the testis. Eighteen cDNA samples composed of 16 ovarian tumor specimens, one normal ovarian tissue and one testis tissue sample were analyzed by real-time RT-PCR. mRNA levels were expressed as percentage differences relative to GAPDH (internal standard) and the levels in normal testis (calibrator).

A preliminary *in silico* mRNA expression profile and characterization of gene products identified in this study was undertaken based on the tissue distribution of expressed sequence tags (ESTs) and serial analysis of gene expression (SAGE) tags in the Cancer Genome Anatomy Database (CGAP: http://cgap.nci.nih.gov/) as well as the information contained in the GeneCards database (http://bioinfo.weizmann.ac.il/cards-bin/). Four antigens, KP-OVA-35, -38, -40 and -52 were identified as CT-like antigens that could potentially serve as targets for ovarian cancer immunotherapy. Of four CT-like antigens, KP-OVA-40/MAGE10A was previously reported as a CT antigen (28). To examine expression of the novel CT-like KP-OVA-35, -38 and -52, conventional RT-PCR was performed using mRNA from normal tissues. These three antigens were expressed strongly only in normal testis (Fig. 1A). The mRNA expression profiles of the KP-OVA-35 and KP-OVA-38 were analyzed in various cancer cell lines and malignant cancer tissues by RT-PCR. Transcripts encoding KP-OVA-35 and -38 were not detected in cancer cell lines and tumor tissues (data not shown). Therefore, KP-OVA-35 and -38 antigens are a virtual CT antigen, as its expressions in both normal tissues and cancer cell lines could not be verified by RT-PCR analysis. To examine the distribution of KP-OVA-52 gene expression in detail, conventional and real time RT-PCR were performed from various cancer cell lines including ovarian cancer cell lines. KP-OVA-52 was expressed in 3 out of 9 ovary cancer cell lines (Fig. 1B) and not expressed in other cancer cell lines including breast, colon, hepatoma, lung, renal, sarcoma, and thyroid anaplastic cell line. Also, quantitative real-time RT-PCR demonstrated KP-OVA-52 in one of the 16 ovarian cancer tissues (Fig. 2).

### ADC activates KP-OVA-52 expression in cancer cell lines

**Figure 3:**
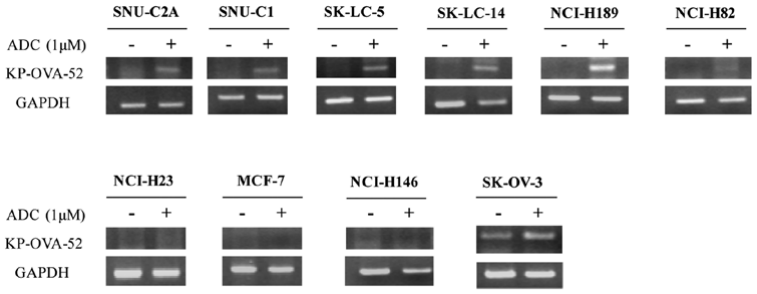
The mRNA expression of KP-OVA-52 in cancer cell lines. Cancer cell lines were treated for 5 days with or without 1 *μ*M ADC. RT-PCR amplifications were performed with primers specific to the KP-OVA-52 gene. GAPDH was used as a loading control for RT-PCR. The ten cell lines used were nine KP-OVA-52 non-expressing cell lines (SNU-C1, SK-LC-5, SK-LC-14, NCI-H189, NCI-H82, SNU-C2A, NCI-H23, MCF-7 and NCI-H146) and expressing cell line (SK-OV-3).

Despite the expression of KP-OVA-52 in ovarian cancer cell lines and ovarian tumors, it is expressed with low frequency and is not expressed in most cancer cells. We determined whether silencing of the KP-OVA-52 gene expression in cancer cell lines was mediated by DNA hypermethylation. For this purpose, we utilized a methylation-specific PCR program (www.urogene.org/methprimer/index1.html) to determine the methylation status of the CpG island in the promoter region of KP-OVA-52. Ten cancer cell lines were treated with 1 *μ*M of ADC for 5 days, and the mRNA expression levels of KP-OVA-52 were analyzed. As demonstrated in Fig. 3, KP-OVA-52 non expressing six cell lines treated ADC demonstrated KP-OVA-52 mRNA expression, while KP-OVA-52 mRNA was not restored expression in three cell lines (NCI-H23, MCF-7 and NCI-H146). In the KP-OVA-52-expressing cell line, SK-OVA-3, KP-OVA-52 mRNA expression with ADC treatment was slightly increased (Fig. 3). These results suggest that the expression of the KP-OVA-52 is activated by the demethylating agent.

### Seroreactivity of isolated antigens by SADA and KP-OVA-52 by western blot analysis

To determine whether immune recognition of these expressed cDNA clones was cancer related, allogeneic sera samples obtained from 20 normal blood donors and 20 patients with ovarian cancer were tested for their reactivity by SADA. SADA is based on the SEREX method and is used to determine seroreactivity against antigens. Each antigen was spotted twice and antigens were only considered positive if the duplicates were positive. A spot was scored as positive if it was clearly darker than the spots corresponding to the negative phage. Of the 75 antigens screened, 13 reacted with a subset of sera from both normal and cancer patients, 14 reacted with two or more allogeneic serum samples from ovarian cancer patients, and the remaining 58 reacted only with the screening sera.

**Figure 4:**
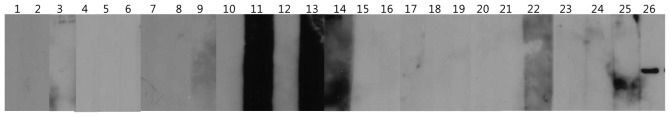
Frequent detection of anti-KP-OVA-52 antibodies in sera from ovarian cancer patients by western blots. Western blots were done with the recombinant KP-OVA-52 protein using 6 normal sera (from lane 1 to lane 6) and 20 ovary cancer sera (from lane 7 to lane 26).

The 17 clones that reacted with sera from both normal and cancer patients or cancer patients alone are listed in Table III. Sera from ovarian cancer patients compared to those from healthy donors showed a high percent reactivity (ranging from 15% to 35%) with 13 antigens (KP-OVA-2, 14, 18, 19, 20, 24, 25, 35, 37, 41, 68, 70 and 73). Of these antigens, four antigens, KP-OVA-25 (TBL2), KP-OVA-35 (TNP1), KP-OVA-68 (C16orf42) and KP-OVA-73 (S100A4) reacted with 20%, 30%, 15%, and 15% ovarian cancer sera, respectively, and not with sera from normal individuals. Of the four genes, the S100A4 (KP-OVA-73) has been shown to function as an autocrine/paracrine factor that plays an important role in the aggressive behavior of ovarian carcinoma (29). Although KP-OVA-52 reacted with 1 of 20 ovarian cancer sera (5%) and not with sera from normal individuals (Table I), it is considered a new CT antigen. Moreover, KP-OVA-52 protein expression was also tested in 20 ovarian cancer sera and six normal sera with western blot analysis. KP-OVA-52 protein expression was only detected in one screening serum and was not detected in normal sera (Fig. 4).

## Discussion

The characterization of tumor-associated antigens, recognized by cellular or humoral effectors of the immune system, represents a new perspective for diagonosis and cancer immunotherapy (20). In this study, SEREX methodology was applied to further definine the spectrum of immunogenic proteins in serous ovarian cancer patients. Immunoscreening of cDNA expression libraries from testis and two ovarian cancer cell lines led to the isolation of 75 independent antigens, designated KP-OVA-1 through KP-OVA-75. The 75 antigens identified in this SEREX analysis of ovarian cancer represent a broad spectrum of cellular components, with 66 being the products of known genes and 9 representing uncharacterized gene products. A striking feature of these antigens was the diversity of gene products recognized by the immune system of ovarian cancer patients, i.e., transcription factors, mRNA splicing factors, translation factors, DNA binding proteins, metabolic enzymes, molecular chaperones, signaling molecules, cytoskeleon proteins and membrane-associated proteins. However, the majority of these SEREX-defined antigens have no known association with cancer or with autoimmunity. CT antigens are immunogenic proteins expressed in normal testis and different type of tumors. Therefore, CT antigens are promising candidates for cancer immunotherapy and the identification of novel CT antigens is a prerequisite for the development of cancer vaccines (20). On the basis of digital expression analysis, four tissue-restricted antigens were identified KP-OVA-35 (TNP1), KP-OVA-38 (C10orf62), KP-OVA-40 (MAGEA10), and KP-OVA-52 (C15orf60).

MAGEA10 is a highly immunogenic member of the MAGEA gene family located in the q28 region of chromosome X and belongs to the CT antigen family. The MAGE-A10 gene has two splice variants which encode the same protein (30) and is expressed by different histological types of tumors, such as non-small cell lung cancer, urothelial carcinoma, head and neck squamous cell carcinoma, hepatocellular carcinoma and melanoma (31,32,33).

Transition protein-1(TNP1, KP-OVA-35) is a spermatid-specific product of the haploid genome which replaces histone and is itself replaced in the mature sperm by the protamines (34). TNP1 encodes a small peptide protein (55 amino acids) that constitutes one exon. The TNP1 gene regulates conversion of nucleosomal chromatin to the compact, non-nucleosomal form found in the sperm nucleus. TNP1 is associated with the appearance of a small set of basic chromosomal transition proteins in the elongating spermatids of mammals. Two uncharacterized proteins, C10orf62 and C15orf60, encode 223 and 266 amino acids, respectively, and the *in vivo* function of these genes is not yet known.

Of these four antigens, our studies focused on KP-OVA-35, -38 and -52. Conventional RT-PCR demonstrated strong mRNA expression in normal testis alone from the three antigens of interest (Fig. 1). However, transcripts encoding KP-OVA-35 and -38 were not detected in cancer cell lines and tumor tissues (data not shown). KP-OVA-52 was expressed in 3 out of 8 ovarian cancer cell lines and with low frequency in ovarian cancer tissues (1/16), but the gene was not expressed in various other cancer cell lines. These results suggest that KP-OVA-52 is likely to be ovarian cancer specific.

For many CT antigens, especially those encoded by the X chromosome (CT-X antigens), expression is regulated by epigenetic mechanisms such as hypermethylation of CpG islands within the promoter region (35). Recently, it has been reported that treatment of ovarian cancer cell lines with ADC increased the expression of the CT antigens tested, including MAGE-A1, MAGE-A3, MAGE-A4, MAGE-A6, MAGE-A10, MAGE-A12, NY-ESO-1, TAG-1, TAG-2a, TAG-2b, and TAG-2c. Thus, treatment with ADC may provide a new therapeutic strategy for modulating CT antigen expression in combination with immunotherapeutic approaches (36).

Using a methylation-specific PCR program (www.urogene.org/methprimer/index1.html) CpG island in the promoter region of KP-OVA-52 was identified. Thus, we investigated whether silencing of the KP-OVA-52 gene expression was mediated by hypermethylation. We found evidence that the expression of KP-OVA-52 is induced by hypomethylation after treatment with the demethylating agent ADC in cancer cell lines not expressing KP-OVA-52 (Fig. 3). Our results clearly indicate a significant correlation between the expression of KP-OVA-52 and hypomethylation.

With regard to immunogenicity, we analyzed anti-IgG antibodies in sera from normal and ovarian cancer patients against the SEREX defined antigens using SADA. Fifty-eight of the 75 antigens were reactive with the screening sera; 13 of these were reactive with sera from both normal donors and cancer patients, and 4 other antigens (KP-OVA-25/TBL2, KP-OVA-35/TNP1, KP-OVA-68/C16orf42 and KP-OVA-73/S100A4) reacted exclusively with sera from cancer patients (ranging from 15% to 30%) (Table III). Despite no expression of the TNP1 gene (KP-OVA-35) in ovarian tumors and several cancer cell lines, TNP1 demonstrated a high percent reactivity (30%). S100A4, which plays an important role in the aggressiveness of ovarian carcinoma cells, was detected in 3 out of 20 allogenic sera. Although results of these two antigens were interesting, a broader analysis will need to be performed in order to determine whether these antigens act as immunogens in ovarian cancer patients. Interesting, KP-OVA-52, considered a new CT antigen, reacted with 1 of 20 ovarian cancer sera by SADA and with 1 of 20 ovarian cancer sera by western blot analysis.

In summary, the current SEREX analysis of ovarian cancer led to the isolation of 4 tissue-restricted gene products, including one known CT antigen (KP-OVA-40/MAGEA10). Two of these differentially expressed antigens (KP-OVA-38/C10orf62 and KP-OVA-52/C15orf60) are novel gene products, and the remaining tissue-restricted antigen (KP-OVA-35/TNP1) has not been previously studied in relation to cancer. Their tissue restricted expression profile and immunogenicity indicate that these four antigens should be further analyzed with regard to their immunotherapeutic potential. Of particular interest is KP-OVA-52, which represents a recently defined CT antigen expressed exclusively in normal testis as well as ovarian cancer. Unlike several other CT antigens, KP-OVA-52 is expressed with low frequency in ovarian cancer and is not expressed in most other cancer cells. Our results demonstrate that the silencing of KP-OVA-52 expression is restored by the demethylating agent ADC and the expression of KP-OVA-52 is suppressed by hypermethylation. Thus, we suggest that the KP-OVA-52 is a CT antigen and may be useful as a potential target for cancer immunotherapy.

## Figures and Tables

**Table I:** Antigens identified through the screening of testis with individual serum.

				Seroreactivity	
KP-OVA-number	Genes (Unigene cluster)^a^	Serum source (no.)	Previously identified by SEREX^b^	Normal (20)	Ovarian cancer patients (20)
1	ZNF234(Hs.235992)	14547	Y	0/20	1/20
2	BRAP(Hs.530940)	14547	Y	3/20	5/20
3	MYNN(Hs.507025)	14547	N	0/20	1/20
4	GARS(Hs.404321)	14547	N	0/20	1/20
5	TXNRD1(Hs.690011)	14547	Y	0/20	1/20
6	PRPF38B(Hs.342307)	14547	Y	0/20	1/20
7	ZFP95(Hs.110839)	14547	Y	0/20	1/20
8	TEKT3(Hs.414648)	14547	N	0/20	1/20
9	RBPJ(Hs.479396)	14547	Y	0/20	1/20
10	GNL2(Hs.75528)	14547	Y	2/20	1/20
11	EID3(Hs.659857)	14547	Y	0/20	1/20
12	RPL26(Hs.644794)	14547	Y	0/20	1/20
13	RPGR(Hs.61438)	14547	N	0/20	1/20
14	PAFAH1B1(Hs.77318)	14547	Y	2/20	7/20
15	PRM1(Hs.2909)	16100	Y	0/20	1/20
16	RANBP5(Hs.712598)	16100	N	0/20	1/20
17	FNBP1(Hs.189409)	16100	Y	0/20	1/20
18	MRPL45(Hs.462913)	16100	Y	1/20	5/20
19	PIK3R3(Hs.655387)	16100	Y	2/20	6/20
20	TEX2(Hs.175414)	16100	N	1/20	6/20
21	TMBIM1(Hs.591605)	16100	Y	0/20	1/20
22	RPL3(Hs.119598)	16100	Y	0/20	1/20
23	GTF2H1(Hs.577202)	16100	N	0/20	1/20
24	GART(Hs.473648)	16100	N	1/20	3/20
25	TBL2(Hs.647044)	16100	N	0/20	4/20
26	AHNAK(Hs.502756)	16100	N	0/20	1/20
27	RPS10(Hs.645317)	16086	Y	0/20	1/20
28	FLJ21228 fis(Hs.677287)	16086	Y	0/20	1/20
29	STXBP3(Hs.530436)	16086	N	0/20	1/20
30	PARP2(Hs.409412)	16086	N	0/20	1/20
31	GKAP1(Hs.522255)	16086	N	0/20	1/20
32	CD164(Hs.520313)	16086	N	0/20	1/20
33	CSNK1D(Hs.631725)	16086	Y	0/20	1/20
34	PDHX(Hs.502315)	16086	Y	0/20	1/20
35	TNP1(Hs.3017)	16086	Y	0/20	6/20
36	CCDC104(Hs.264208)	16086	N	0/20	2/20
37	TPT1(Hs.374596)	16086	Y	2/20	6/20
38	C10orf62(Hs.662302)	16160	N	0/20	1/20
39	KIF27(Hs.697514)	16160	N	0/20	1/20
40	MAGEA10(Hs.18048)	16160	N	0/20	1/20
41	BPTF(Hs.444200)	16160	N	1/20	6/20
42	SETDB2(Hs.631789)	16160	Y	0/20	1/20
43	C16orf80(Hs.532755)	16160	N	0/20	1/20
44	JAKMIP2(Hs.184323)	16160	Y	0/20	1/20
45	CREM(Hs.200250)	16160	N	0/20	1/20
46	FAM128B(Hs.469925)	16160	Y	0/20	1/20
47	ZBTB44(Hs.178499)	16160	N	0/20	1/20
48	LRRC6(Hs.591865)	16160	N	0/20	1/20
49	HSF2(Hs.158195)	16160	Y	0/20	1/20
50	USP1(Hs.35086)	16160	Y	0/20	1/20
51	EIF4G3(Hs.467084)	16160	Y	0/20	1/20
52	C15orf60(Hs.730877)	16160	Y	0/20	1/20

a Unigene cluster of isolated antigens (http://www.ncbi.nim.nih.gov/);

b sequences were compared with those contained in the SEREX database of the Ludwig Institute for Cancer Research (http://ludwig-sun5.unil.ch/CancerImmunomeDB/). Y and N, indicate whether the antigen matched or did not match to an antigen in the SEREX database, respectively.

**Table II:** Antigens identified through the screening of ovarian cancer cell lines with individual serum.

				Seroreactivity	
KP-OVA-number	Genes (Unigene cluster)^a^	Source of library and serum	Previously identified by SEREX^b^	Normal (20)	Ovarian cancer patients (20)
53	KRT17(Hs.2785)	SNU840/no.14495	Y	0/20	1/20
54	HMGA1(Hs.518805)	SNU840/no.14495	N	0/20	1/20
55	FAM63B(Hs.591122)	SNU840/no.14495	Y	3/20	1/20
56	NHP2L1(Hs.182255)	SNU840/no.14495	N	0/20	1/20
57	TK1(Hs.515122)	SNU840/no.14495	N	0/20	1/20
58	DDB1(Hs.290758)	SNU840/no.14495	N	0/20	1/20
59	NME1(Hs.463456)	SNU840/no.14547	Y	0/20	1/20
60	OGFR(Hs.67896)	SNU840/no.14547	Y	0/20	1/20
61	RPL14(Hs.730621)	SNU840/no.14547	Y	0/20	1/20
62	EIF3S5(Hs.516023 )	SNU840/no.14547	N	0/20	1/20
63	Hist1h1c(Hs.7644)	SNU840/no.14547	Y	0/20	1/20
64	GUK1(Hs.376933)	SNU840/no.14547	N	1/20	1/20
65	HOMER3(Hs.720208)	SNU840/no.14547	N	0/20	1/20
66	GSDMD(Hs.118983)	SNU840/no.14547	Y	0/20	1/20
67	KIF7(Hs.513134)	SK-OV-3/no.16160	N	0/20	1/20
68	C16orf42(Hs.134846)	SK-OV-3/no.16160	Y	0/20	3/20
69	TINP1(Hs.482526)	SK-OV-3/no.16160	N	0/20	1/20
70	SSNA1(Hs.530314)	SK-OV-3/no.16160	Y	1/20	5/20
71	FTH1(Hs.524910)	SK-OV-3/no.16160	N	0/20	1/20
72	TSPAN4(Hs.654836)	SK-OV-3/no.16160	Y	0/20	1/20
73	S100A4(Hs.654444)	SK-OV-3/no.16160	Y	0/20	3/20
74	PRP6(Hs.31334)	SK-OV-3/no.16160	Y	0/20	1/20
75	MRPS26(Hs.18946)	SK-OV-3/no.16160	Y	0/20	1/20

a Unigene cluster of isolated antigens (http://www.ncbi.nim.nih.gov/);

b sequences were compared with those contained in the SEREX database of the Ludwig Institute for Cancer Research (http://ludwig-sun5.unil.ch/CancerImmunomeDB/). Y and N, indicate whether the antigen matched or did not match to an antigen in the SEREX database, respectively.

**Table III:** Antigens with high seroreactivity.

		Seroreactivity (%)	
KP-OVA-number	Genes (Unigene cluster)	Normal	Ovarian cancer patients
2	BRAP(Hs.530940)	15	25
14	PAFAH1B1(Hs.77318)	10	35
18	MRPL45(Hs.462913)	5	25
19	PIK3R3(Hs.655387)	10	30
20	TEX2(Hs.175414)	5	30
24	GART(Hs.473648)	5	15
25	TBL2(Hs.647044)	0	20
35	TNP1(Hs.3017)	0	30
36	CCDC104(Hs.264208)	0	10
37	TPT1(Hs.374596)	0	30
41	BPTF(Hs.444200)	5	30
68	C16orf42(Hs.134846)	0	15
70	SSNA1(Hs.530314)	5	25
73	S100A4(Hs.654444)	0	15

The ovarian cancer antigens are with high seroreactivity to both normal donors and ovarian cancer patients or to ovarian cancer patients alone.
